# Cancer-Derived Extracellular Vesicle-Associated MicroRNAs in Intercellular Communication: One Cell’s Trash Is Another Cell’s Treasure

**DOI:** 10.3390/ijms20246109

**Published:** 2019-12-04

**Authors:** Joseph Mills, Marina Capece, Emanuele Cocucci, Anna Tessari, Dario Palmieri

**Affiliations:** 1 Department of Cancer Biology and Genetics, College of Medicine and Comprehensive Cancer Center, The Ohio State University, Columbus, OH 43210, USA; Joseph.Mills@osumc.edu (J.M.); Marina.Capece@osumc.edu (M.C.); Anna.Tessari@osumc.edu (A.T.); 2 Division of Pharmaceutics and Pharmacology, College of Pharmacy and Comprehensive Cancer Center, The Ohio State University, Columbus, OH 43210, USA; Emanuele.Cocucci@osumc.edu

**Keywords:** miRs, microRNAs, extracellular vesicles, EVs, exosomes, microvesicles, tumor microenvironment

## Abstract

Several non-protein-coding genomic regions, previously marked as “junk DNA”, have been reported to be transcriptionally active, giving rise to non-coding RNA species implicated in fundamental biological and pathological processes. In particular, microRNAs (miRNAs), a class of small non-coding RNAs mediating post-transcriptional gene silencing, are causally involved in several human diseases, including various cancer types. Extracellular vesicles (EVs) are membranous structures physiologically released by most cell types. Initially, they were considered a “waste-removal” mechanism, through which cells could dispose unnecessary material and organelles. It is now widely demonstrated that EVs also play a critical role in intercellular communication, mediating the horizontal transfer of lipids, proteins, and genetic material. A paradigm shift in the biology of miRNAs was represented by the discovery that EVs, especially from cancer cells, contain miRs. EV-associated miRs act as autocrine, paracrine and endocrine factors, participating in cancer pathogenesis by modulating intercellular communication. Noteworthy, these formerly neglected molecules are now considered the next generation of cancer “theranostic” tools, with strong clinical relevance. In this review, we aim to summarize the most recent findings regarding EV-associated miRs in cancer pathogenesis and in the development of novel anti-neoplastic diagnostic and therapeutic approaches.

## 1. Introduction

MicroRNAs (also known as miRNAs or miRs) are a class of small (~20–25 nucleotides, nt) non-coding RNAs, initially identified as post-transcriptional regulators of gene expression [1,2,3,4]. miRNAs have been identified in almost all eukaryotic species, supporting their evolutionarily relevant biological role [5]. To date, more than 2600 miRNAs have been identified in humans [6], and the majority of human protein-coding mRNAs are predicted to be regulated by one or more miRNAs [7]. 

Deregulation of miRNA functions and levels are associated to numerous human diseases. Initially, this observation was related to their ability to regulate the expression of most human genes and the activity of critical biological processes such as cell proliferation, differentiation, and apoptosis [8,9]. Several studies have demonstrated that miRNA dysregulation plays a causal role in multiple steps of cancer pathogenesis. In fact, based on their ability to modulate oncogenic or tumor-suppressive gene networks [8] (in a cell context-dependent manner), several miRNAs can be considered tumor suppressors or oncogenes, respectively. Moreover, over the years, several tumor-specific miRNA signatures have been identified, demonstrating that differential miRNA expression patterns are potentially useful diagnostic, prognostic, and predictive biomarkers [10].

A first major paradigm shift was reported in 2007, when Valadi and colleagues demonstrated that miRNAs are also present in biological fluids, both as circulating free miRNAs (cfmiRNAs) and associated with membranous lipid structures, named extracellular vesicles (EVs) [11]. This report opened avenues to a multitude of studies focused on EV-associated miRNAs.

In this review, we aim to summarize some of the most relevant information about the biology of EV-associated tumor miRNAs and their role in intercellular communication and in cancer pathogenesis. Because of their strong potential as biomarkers, we also report an up-to-date (November 2019) summary of clinical trials including EV-associated miRNAs. Finally, we describe some of the most recent and relevant technologies based on EVs and miRNAs, currently being pre-clinically or clinically developed as anti-neoplastic therapeutic agents.

### 2. miRNA Biogenesis

**Figure 1:**
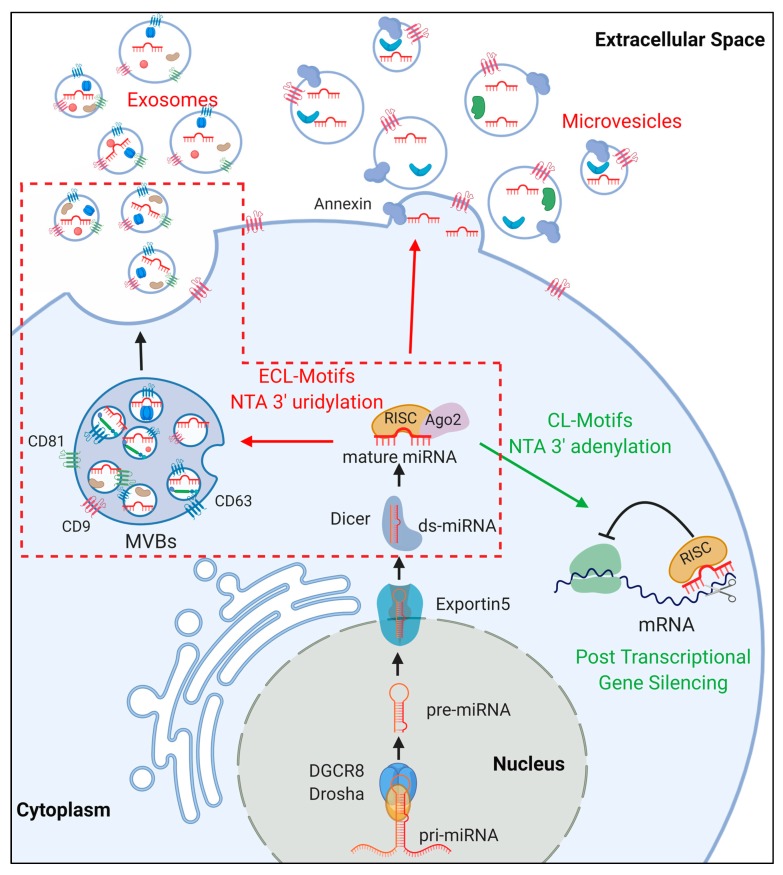
MicroRNA (miRNA) biogenesis and sorting in extracellular vesicles. Upon processing (see text), mature miRNAs are either retained in the cytoplasm of the producing cell, mediating Post-transcriptional gene silencing, or sorted as cargo in extracellular vesicles (EVs) (exosomes and microvesicles). The molecular players in this sorting process are still not completely identified. Extra-cellular localization (ECL) and Cellular localization (CL) motifs in miRNA sequence, as well as nontemplate terminal nucleotide (NTA 3′) additions are reported to modulate this process. A more detailed description of the miRNA sorting process in multivesicular bodies (MVBs)-exosomes (red dashed box) is reported in Figure 2.

miRNA genes are generally transcribed by RNA Polymerase II as long primary transcripts (pri-miRNAs, >100 nt), that are capped and polyadenilated [12]. In some cases, miRNAs are clustered in the genome and, therefore, they are expressed as single polycistronic RNAs. In other cases, miRNAs are located in intronic regions (mirtrons), and their expression is related to that of their host gene [12]. In their canonical biogenesis, pri-miRNAs are recognized by the microprocessor complex, which includes the RNase III enzyme Drosha and the DiGeorge Critical Region 8 (DGCR8) RNA binding protein [13] (Figure 1). Pri-miRNAs display characteristic stem-loop structures that are required for their interaction with and successive cleavage by the microprocessor complex [14]. The microprocessor complex releases a shorter stem-loop precursor miRNA (pre-miRNA), which is exported to the cytoplasm by the Exportin-5 nuclear transporter [15]. In humans, the RNase III-type enzyme Dicer and the trans-activation responsive RNA binding protein TRBP collaborate in the formation of a 20–25 nt dsRNA by cleaving the pre-miRNA loop and 5′/3′ nucleotides [16]. One of the two RNA strands (mature miRNAs) is then loaded into the RNA-induced Silencing Complex (RISC), which includes members of the Argonaute (Ago) protein family. Notably, each strand (generally known as 5p and 3p strands) of the precursor miRNA can be loaded in the RISC complex, with a 5p vs. 3p proportion that is dependent on the cellular environment [17]. The miRNA-equipped RISC complex uses the “seed sequence” (nucleotides 2–7 of the mature miRNA) to recognize the 3′ untranslated region (3′UTR) of target mRNAs. Upon target binding, the RISC complex either inhibits mRNA translation [18] or leads to mRNA decay [19], ultimately resulting in post-transcriptional gene silencing.

Although canonical miRNA biogenesis is composed by a series of well-defined molecular events, it has been demonstrated that additional protein factors participate to the recognition of specific classes of miRNAs, displaying still-debated abilities to modulate miRNA processing and biogenesis [5]. Moreover, post-transcriptional modification of canonical biogenesis factors also play a fundamental role in the regulation of miRNA processing and function. An extensive review of these molecular processes was performed by Treiber et al. and Gebert and MacRae [5,20].

## 3. Physiopathology of Extracellular Vesicles (EVs)

### 3.1. Classification of EVs

EVs have been widely reported as integral members of intercellular communication [21,22], despite their early description as mediators of the disposal of unnecessary cellular components [23,24]. EVs are a heterogeneous group of vesicles released almost by all cells [25]. Originally, these vesicles were considered to originate from multivesicular bodies (MVBs). Due to the well-described role of MVBs in the delivery of cellular material to lysosomes, EVs were initially considered responsible for the removal of unnecessary molecules and components from the cell [26]. However, more recent studies have demonstrated that EVs play a critical role in intercellular communication [11,27,28,29,30,31]. In fact, cells communicate with both adjacent and distant cells, using EVs as mediators which allow the delivery of multitudes of molecular cargoes, including proteins, lipids, DNA, and RNA [32,33]. 

EVs are primarily classified into two major distinct categories: exosomes and microvesicles (MVs) [32,34].

Exosomes are small particles composed of a lipid bilayer ranging from ~20 nm up to ~120 nm in diameter. Subtypes of exosomes have been discovered in various laboratories, including exomeres (35 nm diameter), small exosomes (Exo-S) (60–80 nM), and large exosomes (Exo-L) (90–120 nm) [35]. Size is not the only discriminant between these three subtypes of exosomes, as protein content, genetic content, and biogenesis are also variable [36]. This variation contributes to the heterogeneity of EVs. 

MVs are the other main category of EVs, described as structures of 150 nm–1000 nm in diameter. Often, MVs are referred to as shedding vesicles because they are released by parent cells via budding from the plasma membrane (see below, “EV biogenesis” paragraph) [37,38,39]. Recently, larger populations of EVs, such as exospheres (4 μm) [40], migrasomes (>1 μm) [41], and large oncosomes (1–10 μm) [42], have been described. The diversity in these larger populations, again, is based not solely on size, but also composition and hypothesized biogenesis.

EV nomenclature has been widely debated, as classification of specific vesicle populations depends not only on their size, but also on their origin, protein content, and cargo [43,44,45]. Recently, EV standards of study were compiled by the International Society of Extracellular Vesicles (ISEV) in the publication “Minimal Information for studies of EVs 2018 (MISEV2018)” [44]. In this publication, ISEV guidelines provide standardized EV population characteristics, as well as experimental procedures involving their separation and isolation.

In multiple studies, bona fide exosomes have been considered as characterized by the presence of surface markers such as CD63, CD81, and CD9. However, this classification is presumably over-simplified. In fact, small EVs containing CD63 and CD9 have been identified, as well as small EVs containing only CD9. Since CD9 is a protein resident on the plasma membrane, it is most likely a marker of small and large MVs [46]. Due to overlapping characteristics between different classes of EVs, a classification based on the topological origin of EVs should be considered as an alternative. In this case, EVs originating from the intracellular compartment should be called exosomes, whereas those budding from the plasma membrane ectosomes [47,48].

Throughout this review, EVs released upon MVB fusion with the plasma membrane will be referred to as exosomes, and EVs released by outward budding from the plasma membrane will be referred to as MVs. Mixed populations of the two will be referred to as EVs when no classification is necessary or available.

### 3.2. EV Biogenesis

Although many strides have been made to obtain complete understanding of EV biogenesis, especially for exosomes and MVs, these biological mechanisms still remain partially elusive. Mechanisms of EV release and formation are often conserved across all cell types. However, the content and stimuli for release varies, and additional studies are required to identify specific determinants of cell-to-cell communication regulation.

Exosomes originate as intraluminal vesicles (ILV) that bud in the lumen of endosomes, leading to the formation of MVBs (Figure 1). Multiple ILVs bud in a single MVB, which can be considered a storage site for prompt exosome release. ILV budding is mediated by the Endosomal-Sorting Complex Required for Transport (ESCRT) [49]. The mechanism determining which MVB will be directed to the plasma membrane is still unclear, but some evidence suggests that high cholesterol content in MVB membrane is required [50]. Once full maturation has occurred, members of the Rab protein family (Rab35 in oligodendroglial cells [51], Rab27 in cancer cells [52,53]) facilitate the docking of MVBs to the plasma membrane via stabilization of actin filaments. Once stabilized, fusion between the MVB and the plasma membrane is mediated by SNARE (soluble N-ethylmaleimide-sensitive component attachment protein receptor) protein complex including SNAP23, ultimately leading to the release of ILVs, then termed exosomes [53,54,55,56,57].

MV formation occurs by pinching of outward budded protrusion of the plasma membrane (Figure 1) [37,58,59]. Upon release, MVs will either interact with specific target cells or be degraded in the extracellular space, releasing their cargo [37].

Similar to exosomes, many cell types are able to shed MVs, and different reports hypothesize that cargo sorting in MVs is significant and controlled in some manner, but the mechanism of content sorting remains only partially elucidated [58,60,61]. As extensively reviewed by van Niel and colleagues, cargo sorting in EVs is a multistep process, and multiple sorting machineries have been identified so far. In the first phase, membrane-bound proteins and lipids scheduled for extracellular release accumulate in discrete microdomains of plasma- (for MVs) or MVB- (for exosomes) membranes. In the second phase, soluble cargoes, including proteins and nucleic acids, accumulate in these microdomains and actively participate to the formation of the nascent MVs or ILVs. Based on these observations, specific soluble cargoes could be sorted into EVs based on their ability to interact with critical protein and lipid components involved in the early stages of EV biogenesis.

### 3.3. miRNA Sorting in EVs

In a seminal paper, Valadi and colleagues first reported that exosomes, along with their lipid and protein payload, contain a significant amount of genetic information, particularly mRNAs and miRNAs [11]. To date, it is widely demonstrated that RNA molecules packaged as cargo in EVs comprise mRNAs [62], lncRNAs [63], rRNAs [64], circRNAs [65], and miRNAs [11,66]. Compared to parent cells, EVs have a significantly smaller amount of RNA; however, the RNA present is predominantly constituted by miRNAs [67]. Specifically, miRNAs found in EVs from many different cell types overlap, suggesting that certain miRNAs are selectively packaged in EVs [68]. Multiple databases, such as miRandola (http://mirandola.iit.cnr.it), Vesiclepedia (http://microvesicles.org), and ExoCarta (http://www.exocarta.org/), collect experimental evidences regarding secreted miRNAs. For the rest of this review, miRNAs within exosomes will be referred to as exomiRNAs (Figure 1).

Specific roles of miRNAs associated to EVs in intercellular communication during physiological processes were extensively reviewed by Yàñez-Mó et al. [69] and Mori et al. [70]. miRNAs secreted in EVs are protected by the enzymatic activity of RNases in the blood and other biological fluids, and for this reason they can reach adjacent and distant cells. Physiological functions of EV-contained miRNAs include modulation of immune response [71], muscle differentiation [72], and metabolic homeostasis in adipose tissue, pancreas, cardiovascular system and central nervous system (reviewed in Mori et al.) [70].

**Figure 2:**
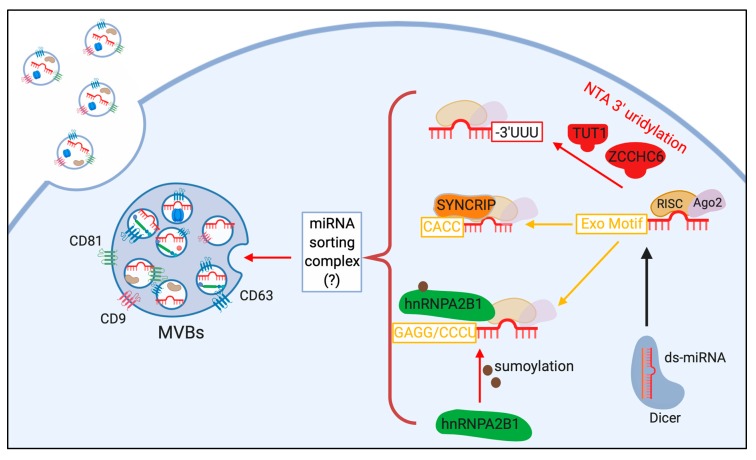
Schematic of currently known mechanisms of miRNA sorting into exosomes. Following processing and association with Ago2 and the RISC complex, specific post-transcriptional events or miRNA sequence influence exosomal sorting. Nontemplate terminal nucleotide additions (NTA) 3′ uridylation of miRNAs is mediated by TUT1 or ZCCHC6. Specific extracellular localization motifs (Exo Motifs) allow protein recognition and exosome sorting. An Exo Motif sequence of CACC is required for SYNCRIP-mediated sorting, while GAGG/CCCU is required for sumoylated hnRNPA2B1-mediated sorting. Figure also reports the hypothesized “miRNA sorting complex” responsible to the final association of miRNAs with MVBs.

Selective sorting of miRNAs in smaller EVs may be an ATP-dependent active phenomenon [73], currently representing a hot topic of investigation. Accordingly, many groups have reported that finely tuned cellular pathways play a role in the modulation of miRNA packaging resulting in intercellular communication, as described below (see Figure 2).

It has been reported that ESCRT knockdown does not affect miRNA sorting, regardless of its role in exosome formation within MVBs [74]. This indicates that selective sorting of miRNAs is regulated by a mechanism independent of exosome biogenesis. Conversely, selective miRNA packaging into exosomes may depend on differential protein expression in multiple cell types, as groups have reported that specific proteins are needed to package miRNAs into exosomes [75,76,77,78,79]. These mechanisms still remain partially elusive due to confounding results obtained using different purification approaches.

Members of the RISC were initially hypothesized as possible players in selective miRNA sorting. In fact, density gradient purification experiments co-identified MVB markers and proteins involved in miRNA-mediated post-transcriptional silencing, such as Ago2 and GW182 [80]. Moreover, several groups have reported that KRAS-MEK signaling is able to regulate Ago2-miRNA loading into exosomes [81,82]. However, in a very recent study, Jeppesen et al. demonstrated that classical exosomes do not contain Ago1–4 proteins or other nuclear or cytoplasmic component of the miRNA machinery (i.e. Drosha, DGCR8, Dicer) [83].

Other groups have been able to demonstrate that specific RNA binding proteins (RBPs) mediate miRNA sorting in cancer cells [75,78,79]. Specifically, several RBPs, such as Synaptotagmin Binding Cytoplasmic RNA Interacting Protein (SYNCRIP) [76], Y-box 1 [75], the heterogeneous nuclear ribonucleoprotein A2B1 (hnRNPA2B1) [78], and major vault protein (MVP) [84] are both able to actively participate in sorting of miRNAs into exosomes, as well as to be sorted alongside their selected miRNAs. In a recent study, Statello et al. (2018) have identified a subset of 30 RBPs present in EVs. These RNA binding proteins may only be present in EVs due to their ability to sort genetic information, like miRNAs, into EVs [85].

Specific sequence motifs present in miRNAs facilitate their interaction with RBPs, resulting in their selective sorting. Villaryoa-Beltri and colleagues reported the identification of exosomal-localization miRNA motifs (e.g., GAGG and CCCU sequences outside the miRNA seed sequence and close to the 3′-end). They further demonstrated that the heterogeneous nuclear ribonucleoprotein A2B1 (hnRNPA2B1) interacts with miRNAs during their packaging in MVBs. Notably, the interaction between exomiRNAs and hnRNPA2B1 was affected by protein post-translational modification, such as sumoylation [78]. A different exosomal-localization motif (GGGU) was identified by Santangelo and colleagues as necessary for the interaction with the RBP SYNCRIP, which, in turn, mediates exosomal localization of these miRNAs (Figure 2) [76].

Post-transcriptional modifications of miRNAs have also been reported as potentially relevant in selective miRNA sorting into exosomes. In fact, Koppers-Lalic et al. demonstrated that 3′-modifications of miRNAs such as uridylation, through a process named “nontemplate terminal nucleodie addition” (NTA), lead to exosomal packaging. The reported factors known to carry out this process are Terminal Uridylyl Transferase 1 (TUT1) and Zinc finger CCHC domain containing 6 (ZCCHC6) proteins [86]. Conversely, intracellular-retained miRNAs displayed increased 3′ adenylation through the same process (Figure 2) [87].

Cellular levels of ceramide also regulate miRNA content in exosomes. In fact, inhibitors of the neutral sphingomyelinase-2 (nSMase2) have been widely used to reduce the quantity of cell-secreted exosomal exomiRNAs. However, the molecular role of ceramide in miRNA sorting in exosomes remains unknown, although potential effects on lipid homeostasis (mediated by nSMase2-inhibitors) could play a role in this phenomenon [74].

Until very recently, miRNA sorting in large EVs, such as MVs, was considered a non-selective process [88]. However, Lee et al. (2019) have demonstrated that Caveolin-1, a lipid raft protein, is able to regulate miRNA sorting into MVs. In response to oxidative stress, phosphorylated Cav-1 (pCav-1) interacts with hnRNPA2B1. Following this interaction, *O*-GlcNacylation of hnRNPA2B1 promotes selective miRNA binding. Upon complex formation, pCav-1 directs the complex to MVs. Ultimately, MVs containing the hnRNPA2B1/miRNA complex are received by macrophages, and stimulate macrophage M1-associated gene expression [77].

All together, these observations strongly support the idea that the sorting of cellular miRNAs into EVs is a tightly regulated process, controlled by a multitude of players and biological pathways. 

For this reason, it is safe to speculate that pathological alterations, such as those observed in cancer, could affect the types and quantity of miRNAs sorted into EVs, resulting in altered miRNA-mediated intercellular communication.

Much like normal cells, cancer cells are able to release EVs and mediate communication among themselves and with other cell types, often to enhance cancer progression [89,90,91]. However, the total number of EVs released by cancer cells is significantly higher than their normal counterparts [92,93]. This observation is often explained by cancer specific activation of multiple oncogenic signaling pathways, such as *SRC* [94], *KRAS-MEK* [81,82], and *H-RAS* [95], as well as overexpression of molecular players involved in membrane fusion machinery, such as PKM2 [96], also play a key role in the positive modulation of EV release. Moreover, different cellular conditions are experienced by cancer cells in comparison with their normal counterparts. Stressful conditions, such as hypoxia, nutrient starvation, or pH changes of the microenvironment are able to enhance the release of EVs and of EV-associated miRNAs.

Studies have demonstrated that cancer cells take advantage of EVs as a disposal mechanism to remove tumor-suppressive miRNAs, such as miR-23b and miR-202-3p, from the cytoplasm [97,98]. However, what makes EV-associated miRNAs relevant in cancer biology and genetics is their critical roles in intercellular communication. By acting as “messengers” between cancer cells and other cellular players of the local and distant microenvironment, EV-associated miRNAs promote cancer cell proliferation, metastatic potential, and resistance to anti-neoplastic treatments. For this reason, studies regarding miRNA-mediated intercellular communication have led to a better understanding of critical mechanisms of cancer pathogenesis.

## 4. Extracellular Vesicles (EVs)-Associated miRNAs as Modulators of Tumor Microenvironment (TME)

Technological developments allowed the identification of circulating miRNAs (both freely circulating and EV-associated) as valid biomarkers of cancer development. However, the specific mechanisms underlying miRNA secretion strongly indicate that their release in the extracellular space is not a mere cancer-associated epiphenomenon.

**Figure 3:**
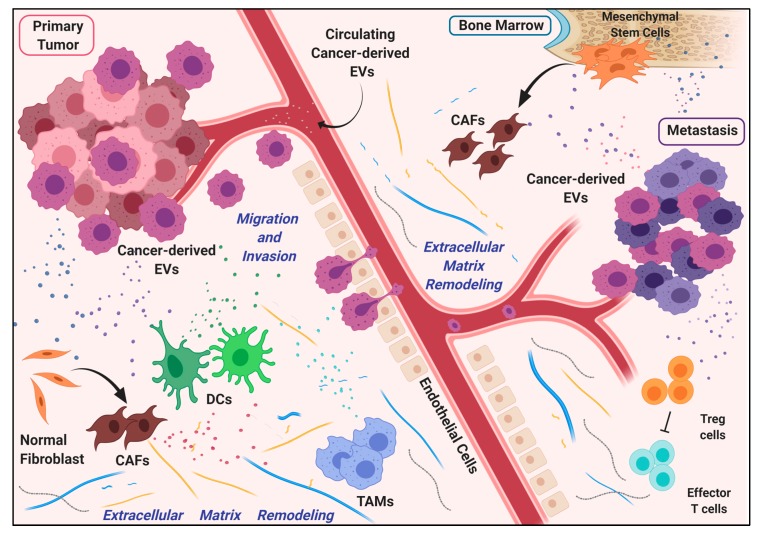
Diagram of the contribution of EVs in metastasis formation. EVs are released by cancer cells from the primary neoplastic lesion, to intensely modify the local and the distant environment. Messages carried in exosomes, such as miRNAs, reshape the extracellular matrix through the activity of Cancer-Associated Fibroblasts (CAFs), Dendritic Cells (DCs) and Tumor-Associated Macrophages (TAMs), preparing favorable conditions for the growth of metastatic cancer cells. Survival and proliferation of cancer cells in the metastatic niche are also supported by mesenchymal and immune cells adequately educated by cancer-derived EVs.

In fact, as small packages with defined and intentionally selected content, EVs represent the perfect tool used by cancer cells of primary neoplastic lesions to alter the local tumor microenvironment (TME), promoting optimal conditions for tumor growth and local invasion. This process ultimately leads to the recruitment and differentiation of cellular components (e.g., cancer associated fibroblasts and mesenchymal stem cells), which participate in the remodeling of TME and support cancer progression. Noteworthy, the reprogramming of TME mediated by cancer cells frequently results in a change of secretory phenotype of surrounding cells, which ultimately triggers an exomiRNA-mediated positive feedback loop (Figure 3).

Finally, the same EVs can mediate intercellular communication distally as well, through systemic circulation similarly to hormones [99,100], preparing the “soil” of a distal organ for its colonization. In 1889, Paget postulated the idea that metastasis formation arises from a process in which cancer cells actively modify the “soil” microenvironment of a specific healthy organ to make it suitable for the growth of the malignant “seed” [101]. In this multi-step process, cancer cells must face and win many fundamental challenges (migration, extravasation, invasion, proper homing, immune system escaping) before conquering the new “territory”.

Here, we report representative examples of how miRNAs specifically packaged into EVs are sharply exploited by primary tumors to shape local and distant regions to promote tumor growth and enhance metastasis formation.

### 4.1. EV-Associated miRNAs in the Modulation of Vascular Permeability

In order for cancer cells to leave their primary site, they need to take advantage of the circulatory or lymphatic systems. EV-associated miRNAs have been reported to help this process, favoring vascular permeability and neoangiogenesis. Tumor cell-secreted miRNAs promote cancer metastasis by destroying vascular endothelial barriers, as in the case of hepatocellular carcinoma that secretes miR-103. This miRNA is then delivered to endothelial cells (ECs) where it inhibits the expression of Vascular Endothelial Cadherin (VE-Cad), p120-catenin (p120) and Zona Occludens 1 (ZO-1), which abrogates endothelial junction integrity [102]. ZO-1 is also downregulated by miR-105, found in the EVs secreted by spontaneous meningeal metastasis from breast cancer cells and delivered to ECs, thus contributing to the destruction of tight junctions [103]. Similarly, brain metastatic breast cancer cells secrete miR-181c through EVs, which targets Phosphoinositide-Dependent Protein Kinase-1 (PDPK1) in ECs, causing vascular endothelium breakdown and leakiness [104].

EVs are also helping the formation of new tumor-associated vessels. Hypoxia, which frequently occurs in the context of proliferating tumors, has been demonstrated to be one of the driving forces for local TME remodeling [105], and a good stimulus for the production of EVs [106]. Upon exomiR-135b delivery from hypoxia-resistant multiple myeloma cells, angiogenesis is promoted through Hypoxia-Inducible Factor-Factor Inhibiting Hypoxia-inducible factor 1 (HIF-FIH) signaling pathway in ECs [107]. Similarly, hypoxic conditions enhance exosomal level of miR-210 of the human leukemia cell line K562, boosting endothelial tube formation of human umbilical vein endothelial cells in a co-culture system [108]. Moreover, hepatocellular carcinoma cells enhance ECs migration and capillary formation through the delivery of miR-210 [109].

### 4.2. Extracellular Matrix Remodeling via miRNA-Containing EVs

Metastasis formation requires an extensive modification of the physiological environment to adequately accommodating cancer cells. Tumor EV-mediated signals profoundly reshape the extracellular matrix.

Through EVs, multiple types of cancer cells “hijack” the behavior and promote the re-localization of tissue-resident cells, such as fibroblasts, macrophages, and also mesenchymal and bone-marrow derived stem cells. This process results in the remodeling of local and distant stroma, enhancing the likelihood of cancer cells to find optimal conditions for metastasis formation [110]. A large number of evidence supports the fact that miRNAs are involved in the activation of fibroblasts, transforming them into Cancer-Associated Fibroblasts (CAFs) [106]. Pancreatic cancer cells secrete miR-155-containing exosomes, which, once taken up by normal fibroblasts, convert them into CAFs through the inhibition of Tumor Protein p53-Inducible Nuclear Protein 1 (TP53INP1) [111]. In a rat model of metastatic pancreatic adenocarcinoma spontaneously metastasizing to lymph nodes and lung, cancer-secreted EVs are enriched in miR-494 and miR-542-3p. These EVs are recovered in lymph nodes and taken up preferentially by lymph node stroma cells and lung fibroblasts. Here, EV-associated miRNAs regulate the expression of *cadherin-17*, *MAL* (Myelin And Lymphocyte protein) and *TRAF4* (TNR Receptor-Associated Factor 4) genes, leading to the upregulation of matrix metalloproteases, thus facilitating cancer cell migration [112]. Similarly, miR-9 secreted via exosomes from triple-negative breast cancer can be transferred to normal fibroblasts, which increases cell motility [113]. On the other hand, “corrupted” fibroblasts can secrete specific exomiRNAs to facilitate cancer cell migration and invasion potential, as in the case of CAF-derived miR-21 and esophageal tumor cells [114], as well as promote epithelial-mesenchymal transition and aggressive phenotype in breast cancer cells [115].

MiR-21 is also secreted by tumor-associated adipocytes, and it is able to promote motility, invasiveness, and aggressiveness of ovarian cancer cells [116]. Moreover, exomiR-21 is released from mesenchymal stem cells (MSCs), improving proliferation and motility of HGC-27 gastric cancer cells [117]. MSCs are also able to sustain viability and migration of multiple myeloma (MM) cells: MM cells transfer miR146a-containing EVs into MSCs, leading to enhanced cytokine and chemokine release, including C-X-C Motif Chemokine Ligand 1 (CXCL1), interleukin 6 (IL-6), and Monocyte Chemoattractant Protein-1 (MCP-1) [118].

Noteworthy, miRNA-containing EVs are used by cancer cells for metabolic reprogramming of the normal stromal tumor microenvironment. It is well known that even in aerobic conditions, cancer cells tend to favor metabolism via glycolysis rather than the much more efficient oxidative phosphorylation (OXPHOS) pathway (Warburg effect) [119,120]. In order to increase their glucose uptake, tumor cells can secrete and transfer miR-122-contaning exosomes to stromal cells, causing the reduction of the expression of pyruvate kinase M2 (PKM2) and glucose transporter 1 (GLUT1) in these cells, consequently increasing the glucose availability for cancer cells [121]. Additionally, human melanoma derived-exosomes contain miR-155 and miR-210 and deliver them to stromal cells, inducing a switch from OXPHOS to glycolysis (reverse Warburg effect), increasing extracellular acidification [122], which is considered to be among the causes of tumorigenesis [123].

### 4.3. miRNAs in EVs Drive Immune Modulation during Metastasis Formation

In their metastatic process, cancer cells are continuously exposed to the threat of the host immune system. It is well known that, as tumor development progresses, cancer cells become able to not only evade the immune system, but also educate immune cells to support tumor growth and metastasis formation through the establishment of a pro-inflammatory milieu [110,124]. Macrophages, dendritic cells, and T lymphocytes are among the immune cells that cancer cells contact and hijack through EV-mediated miRNA delivery. Non-small lung cancer cells secrete exomiR-21 and -29a, which act like PAMPs (pathogen-associated molecular patterns) signals for tumor-infiltrating macrophages. Thus, these miRNAs bind and activate toll-like receptor (TLR) 7 and 8, which, in turn, mediate the pro-tumoral inflammatory reaction through the secretion of pro-inflammatory cytokines, such as IL-6 and TNF-α [27]. Likewise, neuroblastoma (NBL) cells secrete miR-21, which, once taken up by human monocytes, causes an exomiRNA-mediated positive feedback loop in which human monocytes transfer miR-155 via EVs to NBL cells, ultimately conferring their resistance to chemotherapy. Additionally, NBL cells induce mixed, but mostly pro-inflammatory (M2) polarization of nonpolarized human monocytes [125]. Similarly, lung cancer cells are able to switch the phenotype of tumor infiltrating macrophages into M2 macrophages via EV miR-103a [126].

In the same way, exomiR-203 from colorectal tumor cells could induce the in vivo differentiation of monocytes to M2 macrophages [127].

EVs shed by glioblastoma cells are loaded with miR-21, and captured by resident microglia, inducing its proliferation and consequent neuro-inflammation to ultimately sustain glioma cell growth [128]. Beside the ability to M2-polarize macrophages, several other miRNAs are sorted in EVs and delivered by tumor-associated macrophages to cancer cells, supporting their invasive potential [129].

On one hand, cancer cells use immune system cells to create a pro-inflammatory environment that benefits tumor growth. On the other hand, they are able to manipulate the host immunity in order to achieve the suppression of the anti-cancer immune response [130]. For example, regulatory T cells (Tregs), a subset of CD4^+^ T cells recruited by cancer cells to evade the immune system [131], can be recruited via EV-signals sent out by cancer cells. Nasopharyngeal carcinoma cells shed EVs enriched with miR-24-3p, which influences the differentiation of T cells and their ability to engage Treg cells [132,133]. Similarly, Lewis lung carcinoma cells transfer miR-214 in EVs to T cells, downregulating Phosphate and Tensin Homolog protein (PTEN) and promoting Treg expansion [134].

Cancer cells can weaken anti-tumor immune response, also impairing the functionality of DCs [130]. Pancreatic cancer-derived EVs modulate TLR4 expression in DCs via miR-203, compromising their tumor suppressive response [135]. Moreover, regulatory factor X-associated protein (RFXAP), an important transcription factor for Major Histocompatibility Complex II (MHC) II, is inhibited by miR-212-3p transferred from pancreatic cancer-secreted exosomes, resulting in decreased MHC II expression and finally in the induction of immune tolerance of DCs [136].

Interestingly, exomiRs are also involved in cachexia-related inflammation observed in several cancers. MVs from lung and pancreatic cancer cells, containing miR-21, induce apoptosis of skeletal muscle cells through the engagement of Toll-Like Receptor (TLR) -7 and -8 in a pattern similar to those observed for tumor associated macrophages [137].

### 4.4. Dormancy in the Metastatic Niche Is Induced by miRNAs in Cancer-Associated EVs

Cancer cells that are metastasizing to a new organ can use another expedient to avoid host immunity and survive undisturbed for many years. Once a metastatic cancer cell has reached the so-called pre-metastatic niche, a single cancer cell can enter a temporary dormant stage. During this time, cancer cells are mostly quiescent and exhibit chemotherapy resistance, rendering possible the recurrence of cancer after many years from the successful treatment of the primary lesion [110]. In the bone marrow metastatic niche, MSCs release exosomes containing miR-23b, -127, -197, -222 and -223, which drive breast cancer cells to enter G_0_ phase of the cell cycle, decreasing their susceptibility to drug treatment [138,139]. Bone marrow microenvironment is important also in acute lymphoblastic leukemia (ALL) [140]. ALL cells exposed to primary human bone marrow niche cells, including bone marrow stromal cells (BMSC) and primary human osteoblasts, show a decrease in miR-221 and -222 levels, with an increase of the target protein p27 (CDKN1B), leading to the accumulation of tumor cells in the G_0_ phase and resistance to chemotherapy-induced death [141]. Moreover, in Chronic Myelogenous Leukemia (CML), ECs in the bone marrow niche express high level of miR-126 and supply it to CML cells, likely through EV-mediated miRNA trafficking, causing decreased cell cycling and apoptosis, and increased frequency of dormant leukemia cells [142].

The evidence reported so far undoubtedly indicates that miRNA-containing EVs are important players in each step of metastases formation. With hormone-like ability to mediate intercellular crosstalk via autocrine, paracrine, or distant cell-cell signaling, EVs mold local and distal tumor environment through miRNA delivery, preparing the soil for a life-threatening metastasis. In this regard, it is crucial to deepen the study of how intercellular communication via EVs occurs, and if there is an miRNA signature associated with it that can be used as a biomarker to predict the metastatic potential of tumors.

## 5. Translational Potential of Extracellular Vesicles (EVs)-Associated miRNAs

### 5.1. miRNA-Containing Extracellular Vesicles as Cancer Biomarkers in the Clinical Setting

According to the World Health Organization, a biomarker is “any substance, structure, or process that can be measured in the body or its products and influence or predict the incidence of outcome or disease” [143]. In the clinical management of cancer patients, biomarkers can be used for screening, diagnosis, differential diagnosis (diagnostic biomarkers), guidance in treatment decision through prediction of the tumor response (predictive biomarkers), and through evaluation of the possible outcome in terms of life expectancies (prognostic biomarkers) [144]. Unfortunately, most of the currently available biomarkers for cancer patients lack accuracy, making tumor biopsies still irreplaceable despite their invasiveness and high costs. In recent years, great interest has been directed to the so-called “liquid biopsies”, which could lead to the identification of novel tumor biomarkers in biological fluids [145,146]. Liquid biopsies have the obvious advantage of being easily acquired, with minimal discomfort for the patients. Identified biomarkers can be dosed many times during the course of the disease, giving a real-time picture of what is happening inside the tumor. Among the most novel circulating biomarkers, we recognize: circulating tumor-DNA (ctDNA) and circulating tumor cells (CTC) [146], circulating RNAs [145], and EVs [147]. In this scenario, cfmiRNAs and exomiRNAs represent the ideal candidates for highly specific and sensitive diagnostic, predictive, and prognostic tests. First of all, cancer cells and normal cells express and package into exosomes different sets of miRNAs (as described above). It is well demonstrated that circulating miRNAs can in part reflect their expression profile in the tumor tissue, making possible the definition of specific diagnostic signatures. Second, cfmiRNAs and exomiRNAs can be found in almost all the biological fluids: blood [145,148], saliva [149,150], urine [151,152], feces [153], cerebrospinal fluid [154], and the literature in this regard is boundless. Differential miRNA packaging has also been observed in response to anti-cancer therapies. This phenomenon, which is able to transfer resistance to treatments among cancer cells, has been observed in a wide range of cancers including pancreatic [155], head and neck carcinoma [156], breast [157], acute myeloid leukemia [158], prostate [159], ovarian [160], lung [161], glioblastoma [162], and hepatocellular carcinoma [163].

Moreover, cfmiRNAs display chemical and physical characteristics that make them relatively stable in the bloodstream [164]. ExomiRNAs are abundantly released by cancer cells (compared to their normal counterpart), and thanks to the protective effect of the vesicles, miRNAs are less sensitive to degradation, even in presence of RNase [165]. Exosomes are resistant to freezing and thawing cycles, and the handling and storage of biological fluid samples can be easy even for small centers [166,167].

For these reasons, specific miRNAs associated with EVs are current potential diagnostic, predictive and prognostic biomarkers for cancer patients [168,169,170].

For the purpose of this review, we focused on studies with potential clinical impact by consulting the ClinicalTrials.gov database. In particular, we performed a research of the combination of the terms: cancer, miRNA, vesicles, extracellular, exosome, circulating, blood, urine, urinary, saliva, cerebrospinal fluid (csf), feces, stool, sputum, breath, pleural, and delivery. We then selected the clinical trials that clearly stated the aim of dosing cf- or EV-associated miRNAs in body fluids as cancer biomarkers. The vast majority of the studies are investigating cfmiRNAs, probably due to the relative novelty of the EV field (Supplementary Table S1). Of the 104 clinical trials that we were able to identify, only 10 aim to isolate and characterize EV-associated miRNAs (Table 1). Of those 10 studies, 7 are investigating the role of EV-associated miRNAs as diagnostic, 3 as predictive, and 6 as prognostic biomarkers. Interestingly, 9 out of 10 studies are evaluating EV-associated miRNAs in blood, while only one is investigating them in bile and one in urine samples. All the studies are currently recruiting. For this reason, no results are available to date.

### 5.2. Therapeutic Applications of EV-Associated miRNAs

EVs recently became one of the most interesting topics in nanotechnology research. They display several characteristics that make them ideal candidates for cancer therapy. Among those features we recognize: stability in the blood stream [164], low immunogenicity [171,172], capability to reach distant sites (see above), ability to penetrate the blood-brain barrier [173], and their natural function as biological carriers. EVs can be weaponized with different compounds, such as drugs (for example doxorubicin or paclitaxel) [174,175]\ and nucleic acids, including siRNAs, miRNAs and anti-miRNAs [176,177]. For a comprehensive review on the state of the art about EV in the delivery of therapeutics, we refer to the recent manuscript by Sil et al. [178]. Briefly, there are two main types of EVs that can be used as therapeutic agents: engineered EVs and EV mimetics. The first group is composed by cell-derived EVs, which are then modified in order to implement their biodistribution, reduce their clearance, and implement their anti-neoplastic effect. In particular, they can be adjusted in their content (for example loaded with cytotoxic drugs [175,179,180]), or in their surface antigens (for example, presenting molecules that can specifically recognize tumor cells [181]). One of the main caveats of this approach is represented by the low amount of EVs that are normally produced by mammalian cells, which raises the question of which cells should be used, how feasible are large-scale EV production and purification [182], how to store them without altering their functions, and how to safely and effectively load them [44]. On the other hand, EV mimetics do not entirely derive from cells but are, at least in part, synthetically designed. Thanks to their synthetic nature, EV mimetics are more consistent in size, composition, and cargo, but they might be lacking some natural components of cell-derived EVs that make them more active. In the clinical oncology settings, EVs have already been tested as therapeutic tools, and clinical trials are currently ongoing. For example, the first phase I trial evaluating the safety profile of human dendritic cell-derived exosomes (DEX) loaded with the Melanoma-Associated AntiGen 3 (MAGE-3), in MAGE-3 expressing melanoma patients, showed a good toxicity profile (no adverse events greater than grade 2), some clinical effect in 1/3 of the patients, and an increase in Natural Killer (NK) cells [183]. DEX have also been studied for the treatment of non-small-cell lung carcinoma (NSCLC) in phase I and II clinical trials [184,185]. Even if the latest did not reach the primary endpoint (at least 50% of patients with progression-free survival of at least 4 months), DEX administration confirmed its ability to enhance antitumor immune response through NK cells in NSCLC patients.

Clinical application of EV-associated miRNAs is still in its embryonic phase. Nevertheless, several groups have demonstrated their potential role in preclinical models of cancer. An example is represented by the exomiRNA cytotoxic signal delivered from NK to tumor cells [186]. The fact that activated NK-derived EVs are able to induce caspase-mediated cell death in different cancer cell types (Acute Lymphoblastic Leukemia, Chronic Myeloid Leukemia, Burkitt Lymphoma, breast cancer, melanoma and neuroblastoma cells) thanks to their content in cytotoxic proteins (such as perforin, granulysin, and granzymes A and B) which was already known [172,187,188]. In a recent paper, Neviani et al. demonstrated that NK-mediated killing of neuroblastoma cells is, at least in part, due to the transfer of miR-186. Interestingly, exosomes were able to exert their cytotoxic activity even in presence of TGFβ-1, which is commonly associated with NK inhibition and cancer immune escape [189,190,191]. Authors were also able to demonstrate an in vivo activity of miR-186-loaded anionic lipopolyplex nanoparticles directed against neuroblastoma cells through their coating with anti-GD2, a neuroblastoma marker. These results not only highlight the capability of EV-associated miRNAs to induce cancer cell death, but also provide insights on the feasibility and safety of these type of nanoparticles. From a translational perspective, NK-derived exosomes and EV mimetics might show a significant improvement in terms of adverse events compared to cell-based therapies, possibly by avoiding the occurrence of the potentially life-threatening cytokine release syndrome.

Few clinical trials evaluating the potential of miRNA delivery by EVs have been performed so far. The first phase I trial of a potential first-in-class liposomal miR-34a mimic, namely MRX34, has been published in 2017 [192]. MiR-34a is well characterized for its oncosuppressive role in many tumors (including tumors of lung, breast, and prostate origin) [193]. Preclinical evidences showed its downregulation in cancer, and how its delivery to cancer cells both in vitro and in vivo reduced cell proliferation, invasion and migration [194,195,196,197,198,199,200]. Safety results from the clinical trial showed an acceptable profile when administered with dexamethasone premedication, which was needed due to infusion-related adverse events. Unfortunately, a subsequent phase I study investigating a different administration schedule (5 days on and 2 weeks off instead of twice a week for 3 weeks followed by one week off) resulted in 5 immune-related serious adverse events which caused the termination of the trial (source ClinicalTrials.gov Identifier NCT01829971), and the withdrawal of an already planned trial on melanoma patients (source ClinicalTrials.gov Identifier NCT02862145). Another clinical experience was performed using miRNA-loaded minicells, namely TargomiRs, in patients with recurrent malignant pleural mesothelioma (MPM) [201]. In particular, TargomiRs were loaded with miR16-based mimic miRNA and targeted Epidermal Growth Factor (EGFR). As miR-15 and miR-16 largely demonstrated their oncosuppressive role in several tumors, their reintroduction as miRNA-mimic led to growth inhibition in models of MPM both in vitro and in vivo. The trial reported 5 dose-limiting toxicities (DLT): cardiac ischaemia, cardiomyopathy, infusion-related reaction, non-cardiac pain, and anaphylactoid reaction; adverse events included transient lymphopenia and hypophosphatemia, increased transaminases and alkaline phosphatase serum levels, and adverse cardiac events (ischaemia, electrocardiogram changes, Takotsubo cardiomyopathy).

As we mentioned before, many studies demonstrated that cancer cells can communicate to the TME through EV-miRNAs, resulting in inhibition of the immune response and tumor immune escape [27,125]. From this standpoint, EVs could be considered not only as carriers for anti-cancer therapeutic agents, but also as potential targets themselves. In particular, the pro-tumorigenic effect of EVs could be impaired through three distinct strategies. First, EVs formation can be inhibited, for example, by nSMase2 inhibition or knock-down, as shown in breast [202] and ovarian [203] cancer models, which result in reduction of EV release and decrease of metastatic tumor potential. Potential drawbacks of this strategy are easily foreseeable: EV release is a physiological phenomenon, and its inhibition on normal cells may cause several toxic effects. A second approach to inhibit EVs could be their elimination from the blood stream, which includes both machine-mediated methods and EV removal through antibodies. Attempts have been done with hemofiltration [204], a technique that is free from drug administration-related toxicities, but is still affected by a degree of invasiveness for the patient. The other rational strategy to eradicate EVs from the circulation would be their targeting by monoclonal antibodies. Unfortunately, to the best of our knowledge, a specific target for cancer-derived EVs is still lacking so far. Finally, the third approach to block malignant EVs would be the inhibition of their uptake from recipient cells. For example, glioblastoma cells showed a reduction in exosome uptake after the treatment with inhibitors of heparin sulfate proteoglycans (HSPGs), which are cell surface molecules that act as exosome receptors in those cells [205]. However, in vivo feasibility of this approach remains to be determined.

## 6. Final Considerations

Initially considered a waste disposal system for cells, EVs are now accepted as a valuable source of genetic information both for a better understanding of cancer pathogenesis and the development of diagnostic, predictive, prognostic and possibly therapeutic approaches.

One of the main obstacles toward a complete knowledge of the biological roles of these membranous structures is represented by the current technical hurdles in their isolation and characterization. Thanks to the efforts of the scientific community, and particularly to the ISEV [44], shared parameters were provided to standardize EV classification and biogenic process.

However, the technological improvements obtained in recent years are now questioning the “canonical” definitions of exosomes, MVs and other small and large EVs. The development of high-resolution density gradient fractionation and direct immunoaffinity capture now provide powerful tools for EV study and characterization, further limiting the artifacts obtained when other approaches (e.g., ultracentrifugation, chemical precipitation) are used. In the recent publication from Jeppesen and colleagues, the use of these two technologies shook the foundations of EV and EV-associated miRNA biology. In fact, this work not only sets a new standard for the study of EVs, but also demonstrates that conventional exosomes do not contain neither Argonaute proteins nor other factors previously associated with miRNAs [83]. For this reason, it is clear that future research in the field of EV-associated miRNAs will have to take into account the power of these technological advancements.

Another technical limitation in the study of the physiology of EVs is based on the inadequateness of molecular biology approaches used in other cellular studies. In particular, the use of plasmid vectors and exogenous promoters (e.g., cytomegalovirus, CMV) leads to significantly higher expression of the protein of interest than physiological expression levels. The use of CRISPR/Cas9-based knock-in systems, however, represent a valid possibility to overcome these technical challenges. In fact, gene-editing approaches have already demonstrated their value in imaging endocytotic events, and are likely able to show similar results also in the process of MVs and exosomes biogenesis [206].

Along with the critical need of standardization and of technological advancements, a better knowledge of the molecular and cellular processes governing the biogenesis of miRNA-containing EVs has already shown a great clinical value, and it is expected to improve in the next years. Despite the great interest and potential of the use of engineered EVs and EV mimetics in the clinic, a more comprehensive understanding of how they should be targeted is still needed. Indeed, therapeutic approaches have unfortunately led so far to significant toxicities (immune-related serious adverse events, cardiotoxicity) [190,199], which need to be overcome and solved prior to their wide clinical use.

Current open questions concern the actual biological events required for miRNA sorting as cargoes of EVs. Studies so far indicate that sequence specificity, as well as the ability to undergo post-transcriptional modification and to interact with specific protein factors, might represent the molecular determinants of miRNA-sorting in EVs. However, these factors represent still unsettled issues, as new technologies query previous findings. Furthermore, the molecular events that coordinate miRNA biogenesis and intra-cellular activity (e.g., post-transcriptional gene silencing) with their potential release in the extra-cellular space are still mostly unknown. A better knowledge of the release process could also have significant impact in the clinical setting: in fact, it is possible to speculate that different factors in normal vs. cancer cells are responsible to miRNA packaging, and their identification could pave the way to the targeted anti-neoplastic approaches based on the selective inhibition of EV-miRNAs by cancer cells. This approach could prevent the above-described effects mediated by secreted miRNAs, such as remodeling of the TME, induction of the premetastatic niche and immunomodulatory effects.

The identification of the mechanisms through which EVs specifically interact with their target cells is also a relevant field of research. It is known that EVs from cancer cells display surface proteins, such as integrins, which play a critical role in the establishment of organotropic metastases [207]. For this reason, the identification of cancer-specific EV markers, and of potential approaches to prevent their interaction with target cells, could provide completely new approaches for the treatment of cancer by impairing intercellular communication.

In summary, although initially considered “transcriptional noise”, miRNAs have proven over the years to play a fundamental role in modulating critical biological and pathological processes. Furthermore, several studies demonstrated that miRNAs can be secreted through regulated biological pathways, enhancing cancer pathogenesis by modulating inter-cellular communication. The next frontiers of EV-associated miRNAs will likely involve both the modulation of EV production from cancer cells and the generation of semi-natural/artificial vesicles. These approaches will ultimately allow clinicians to “counteract” the EV-dependent hijacking triggered by tumors, fighting back using the same weapons used by cancer cells.

Based on the studies summarized in this review, we speculate that extracellular miRNAs will participate to revolutionize diagnostic and therapeutic protocols, potentially providing relevant benefits for a wide range of cancer patients.

## Figures and Tables

**Table 1:** Clinical trials listed on clinicaltrials.gov analyzing EV-associated miRNA in body fluids as cancer biomarkers.

Name of the Study	Disease	Phase	miRNA	Sample Source	NCT Identifier
Pathogenic Mechanisms of Cancer and Cardiovascular Diseases	All tumors	NA	exomiRNA	Blood	NCT03051191
U01-Biomarkers for Noninvasive and Early Detection of Pancreatic Cancer	Pancreatic cancer	NA	cfmiRNA, exomiRNA	Blood	NCT03886571
Evaluation of MicroRNA Expression in Blood and Cytology for Detecting Barrett’s Esophagus and Associated Neoplasia	Esophageal cancer	NA	exomiRNA	Blood, bile	NCT02464930
Prostasomes as Diagnostic Tool for Prostate Cancer Detection	Prostate cancer	NA	exomiRNA	Blood	NCT03694483
To Investigate the Diagnostic Accuracy of Exosomal microRNA in Predicting the Aggressiveness of Prostate Cancer in Chinese Patients	Prostate cancer	NA	exomiRNA	Urine	NCT03911999
Non-coding RNA in the Exosome of the Epithelia Ovarian Cancer	Ovarian cancer	NA	exomiRNA	Blood	NCT03738319
Identification and Characterization of Predictive Factors of Onset of Bone Metastases in Cancer Patients (PreMetOn)	Bone metastasis	NA	exomiRNA	Blood	NCT03895216
Neoadjuvant Nivolumab for Oral Cancer Combined With FDG and Anti-PD-L1 PET/CT Imaging for Response Prediction (NeoNivo)	Oral cancer	Phase I	EV-associated miRNA	Blood	NCT03843515
Circulating Exosome RNA in Lung Metastases of Primary High-Grade Osteosarcoma	Osteosarcoma	NA	exomiRNA	Blood	NCT03108677
Study of Exosomes in Monitoring Patients With Sarcoma (EXOSARC)	Sarcoma	NA	exomiRNA	Blood	NCT03800121

Abbreviations: NA, Not Available; FDG, Fludeoxyglucose; PET/CT Positron Emission Tomography–Computed Tomography; PD-L1, Programmed death-ligand 1

## Supplementary Materials

Supplementary materials can be found at https://www.mdpi.com/1422-0067/20/24/6109/s1. Table S1: Clinical trials listed on clinicaltrials.gov analyzing cfmiRs in body fluids as cancer biomarkers.
