# Supplementary material for: Cancer-Derived Extracellular Vesicle-Associated MicroRNAs in Intercellular Communication: One Cell’s Trash Is Another Cell’s Treasure
Source: Int J Mol Sci. 2019 Dec 4;20(24):6109. doi: 10.3390/ijms20246109 (PMC6940802; doi:10.3390/ijms20246109)
Supplement: Supplementary file 1 [file ijms-20-06109-s001.pdf]

Supplementary Table 1. Clinical trials listed on clinicaltrials.gov analyzing cfmiRs in body fluids as cancer biomarkers.

Name of the Study	Disease	Phase	miR	Sample Source	NCT Identifier
Effect of Remote Ischaemic Conditioning in Oncology Patients (ERIC-ONC)	All tumors	NA	cfmiR	Blood	NCT02471885
A Phase 1b Study of Axitinib In Combination with Crizotinib In Patients with Advanced Solid Tumors	Solid tumors	Phase I	cfmiR	Blood	NCT01999972
A Phase 1/2A Study of Minerval in Adult Patients with Advanced Solid Tumours	Solid tumors	Phase I/II	cfmiR	Blood	NCT01792310
Oral Histone Deacetylase Inhibitor 4SC-202 in Patients with Advanced Hematologic Malignancies (TOPAS)	Solid tumors	Phase I	cfmiR	Blood	NCT01344707
Plasma miRs Predict Radiosensitivity of Different Fractionation Regimes in Palliative Radiotherapy for Advanced Non-Small Cell Lung Cancer： Multicenter Controlled Study (RadimiR-01)	NSCLC	NA	cfmiR	Blood	NCT03074175
Papaverine Hydrochloride and Stereotactic Body Radiation Therapy in Treating Patients with Non-small Cell Lung Cancer	NSCLC	Phase I	cfmiR	Blood	NCT03824327
Plasma miR Profiling as First Line Screening Test for Lung Cancer Detection: A Prospective Study (BIOMILD)	NSCLC	NA	cfmiR	Blood	NCT02247453
Early Lung Cancer Detection in High Risk Individuals (MILD)	NSCLC	NA	cfmiR	Blood	NCT02837809
Identification of Predictive and Prognostic Markers for Lung Cancer with Metastases	NSCLC	NA	cfmiR	Blood	NCT02301858
Micro RNA Genetic Signature in NSCLC Egyptian Patients	NSCLC	NA	cfmiR	Blood, BAL	NCT02445924
Blood Samples from Patients with Non-Small Cell Lung Cancer and From Healthy Volunteers	Lung cancer	NA	cfmiR	Blood	NCT00897234
The Effect of Berries on Lung Cancer Tumors	Lung cancer	NA	cfmiR	Blood	NCT00681512
Monitoring the Changes of Tumor-related Biomarkers Before and After Pulmonary Nodule Biopsy	Lung cancer	NA	cfmiR	Blood	NCT03397355
Bronchoscopy Assisted by Electromagnetic Navigation (EMN) in the Diagnosis of Small Pulmonary Nodules	Pulmonary nodule	Phase II	cfmiR	Blood, BAL, exhaled breath	NCT01779388
ATREUS - Phase II Study on the Activity of Trabectedin in Patients with Malignant Pleural Mesothelioma (MPM) (ATREUS)	MPM	Phase II	cfmiR	Blood	NCT02194231

Ramucirumab and Carbo-Paclitaxel for Untreated Thymic Carcinoma / B3 Thymoma With Carcinoma (RELEVENT)	Thymoma	Phase II	cfmiR	Blood	NCT03921671
Adapted Physical Activity (APA) in a Breast Cancer Population.	Breast cancer	NA	cfmiR	Blood	NCT03528473
Carvedilol Effect in Preventing Chemotherapy - Induced Cardiotoxicity (Ceccy)	Breast cancer	Phase III	cfmiR	Blood	NCT01724450
Circulating miRs as Biomarkers of Hormone Sensitivity in Breast Cancer (MIRHO)	Breast cancer	Phase IV	cfmiR	Blood	NCT01612871
STI.VI. Study: How to Improve Lifestyles in Screening Contexts (STIVI)	Breast cancer, Colon cancer	NA	cfmiR	Blood	NCT03118882
Identification and Evaluation of Biomarkers of Resistance to Neoadjuvant Chemotherapy (IDEA SEIN) (IDEASEIN)	Breast cancer	NA	cfmiR	Blood	NCT03255486
Circulating MiR as Biomarker of Cardiotoxicity in Breast Cancer	Breast cancer	NA	cfmiR	Blood	NCT02065908
Circulating miRs. ICORG 10-11, V2	Breast cancer	NA	cfmiR	Blood	NCT01722851
miR and Relevant Biomarkers of BC Patients Undergoing Neoadjuvant Treatment	Breast cancer	NA	cfmiR	Blood	NCT03779022
The Andromeda Study. Predictive Value of Combined Criteria to Tailor Breast Cancer Screening	Breast cancer	NA	cfmiR	Blood	NCT02618538
Imaging Patients for Cancer Drug Selection-Metastatic Breast Cancer (IMPACT-MBC)	Breast cancer	NA	cfmiR	Blood	NCT01957332
REBECCA Study (Radiotherapy for Breast Cancer and Cardiotoxicity) (REBECCA)	Breast cancer	NA	cfmiR	Blood	NCT02079272
Anti-proliferative Effects of Vitamin D and Melatonin in Breast Cancer (MELO-D)	Breast cancer	Phase II	cfmiR	Blood	NCT01965522
Pregnant Breast Cancer: Early/Advanced/Metastatic (PRAEGNANT)	Breast cancer	NA	cfmiR	Blood	NCT02338167
Breast Cancer and Cardiotoxicity Induced by RAdioTherapy: the BACCARAT Study (BACCARAT)	Breast cancer	NA	cfmiR	Blood	NCT02605512
Early Detection of Cardiovascular Changes After Radiotherapy for Breast Cancer (EARLY-HEART)	Breast cancer	NA	cfmiR	Blood	NCT03297346
Timisnar-Biomarkers Satellite Study (Timisnar-miR) (TiMiSNAR-miR)	Rectal cancer	NA	cfmiR	Blood	NCT03962088

Atu027 Plus Gemcitabine in Advanced or Metastatic Pancreatic Cancer (Atu027-I-02) (Atu027-I-02)	Pancreatic cancer	Phase I/II	cfmiR	Blood	NCT01808638
Detection of MiR-25 in the Diagnosis of Pancreatic Cancer	Pancreatic cancer	NA	cfmiR	Blood	NCT03432624
A Prospective Translational Tissue Collection Study in Early and Advanced Pancreatic Ductal Adenocarcinoma and Pancreatic Neuroendocrine Tumours to Enable Further Disease Characterisation and the Development of Potential Predictive and Prognostic Biomarkers (PaC-MAn)	Pancreatic cancer	NA	cfmiR	Blood, urine, feces, saliva, bile	NCT03840460
U01-Biomarkers for Noninvasive and Early Detection of Pancreatic Cancer	Pancreatic cancer	NA	cfmiR, exomiR	Blood	NCT03886571
Validation of Useful Markers Generated by Next Generation Bio-data Based Genome Research and Cohort Study (miR_Chip)	Pancreatic cancer	NA	cfmiR	Blood	NCT02807896
Colorectal Cancer Detected by 1H-NMR Spectroscopy (BIOCOR)	Colon cancer	NA	cfmiR	Blood	NCT02364154
A Biomarker Study in Patients Getting Regorafenib for Metastatic Colorectal Cancer	Colon cancer	Phase II	cfmiR	Blood	NCT02402036
Biomarkers for the Early Diagnosis, Prediction, Prognosis for Colorectal Cancers	Colon cancer	Phase I	cfmiR	Blood	NCT01828918
Predictive and Prognostic Value of Inflammatory Markers and miR in Stage IV Colorectal Cancer	Colon cancer	NA	cfmiR	Blood	NCT04149613
The Effects of Replacing Red and Processed Meat with Quorn on Biomarkers of Gut Health (MYCOMEAT)	Colon cancer	NA	cfmiR	Blood	NCT03944421
ColoCare Study-Colorectal Cancer Cohort	Colon cancer	NA	cfmiR	Blood, urine, feces, saliva	NCT02328677
Prediction Model of Response for CCRT in Esophageal Cancer	Esophageal cancer	NA	cfmiR	Blood	NCT03081988
The Utility of Circulating Tumour Cells and Plasma miR in Esophageal Adenocarcinoma	Esophageal cancer	NA	cfmiR	Blood	NCT02812680
Prediction Model of Response for CCRT in Esophageal Cancer	Esophageal cancer	NA	cfmiR	Blood	NCT03081988
Predicting Biomarker of Gastric Cancer Chemotherapy Response	Gastric cancer	NA	cfmiR	Blood	NCT03253107
Genetic Profiling of Liver Cancer in Patients Undergoing Liver Transplantation	HCC	NA	cfmiR	Blood	NCT02412579
Clinical Significance of Hepatic and Circulating miRs miR-221 and miR-222 in Hepatocellular Carcinoma	HCC	NA	cfmiR	Blood	NCT02928627

Fingerprint Characterization of Sorafenib Treated HCC (HCC-e:Med2)	HCC	NA	cfmiR	Blood	NCT03958669
Study of miRs as a Diagnostic Tool for HCV-related Hepatocellular Carcinoma	HCC	NA	cfmiR	Blood	NCT03429530
miRs as Diagnostic Biomarkers in Hepatocellular Carcinoma Among Somali Patients	HCC	NA	cfmiR	Blood	NCT03227510
Impact of IL-28B rs12979860 and rs4803217 Gene Polymorphisms Associated With miRs Deregulation on HCV-related Hepatocellular Carcinoma	HCC	NA	cfmiR	Blood	NCT02507882
Exploration of Molecular Biomarkers for Lu-177 DOTATATE Therapy in Midgut Neuroendocrine Tumor (GENEBIOLuNET)	Neuroendocrine tumors	NA	cfmiR	Blood	NCT03667092
miR Profiles Identification in Adeno Carcinoma Prostate Cancer	Prostate	NA	cfmiR	Blood	NCT02964351
Evaluating the Impact of Prostate Only Versus Pelvic Radiation for N+ Prostate Cancer. (PROPER)	Prostate cancer	Phase III	cfmiR	Blood	NCT02745587
Personalizing Enzalutamide Therapy by Understanding the Relation Between Tumor mRNAs, miRs and Treatment Response (ILUMINATE)	Prostate cancer	NA	cfmiR	Blood	NCT02471469
Abiraterone Acetate in Treating Patients with Metastatic Hormone-Resistant Prostate Cancer	Prostate cancer	Phase II	cfmiR	Blood	NCT01503229
The Potential Role of miR-155 And Telomerase Reverse Transcriptase in Diagnosis of Non-Muscle Invasive Bladder Cancer and Their Pathological Correlation	Bladder cancer	NA	cfmiR	NA	NCT03591367
Studying Genes in Samples from Younger Patients with Adrenocortical Tumor	Adrenocortical tumors	NA	cfmiR	Blood	NCT01528956
miR Expression in Renal Cell Carcinoma	RCC	NA	cfmiR	Blood, urine	NCT00743054
Study of Hydroxychloroquine Before Surgery in Patients with Primary Renal Cell Carcinoma	RCC	Phase I	cfmiR	Blood	NCT01144169
Cohort of Patients with Pelvic Gynecological Cancer: Constitution of a Collection of Biological Samples with Radioclinic Characterization (PELVIMASS2)	Gynecological cancer	NA	cfmiR	Blood	NCT03622983

Exploring the Potential of Novel Biomarkers Based on Plasma miRs for a Better Management of Pelvic Gynecologic Tumors (GYNO-MIR)	Gynecological cancer	NA	cfmiR	Blood	NCT03776630
Urinary miR in Endometrial Cancer Study	Endometrial cancer	NA	cfmiR	Urine	NCT03824613
Search for Predictors of Therapeutic Response in Ovarian Carcinoma (miRSa)	Ovarian cancer	NA	cfmiR	Blood	NCT01391351
Molecular Mechanisms Leading to Chemoresistance in Epithelial Ovarian Cancer (CHEMOVA)	Ovarian cancer	NA	cfmiR	Blood, urine	NCT02758652
Studying Tissue and Blood Samples from Patients with Acute Myeloid Leukemia	AML	NA	cfmiR	Blood	NCT00900224
Evaluation of ProALL miRs in Blood Specimen for Prediction of ALL Relapse Risk	B-ALL	NA	cfmiR	Blood	NCT03000335
Identification of Hematological Malignancies and Therapy Predication Using miRs as a Diagnostic Tool	Hematological malignancies	NA	cfmiR	Blood	NCT02791217
Establishment of a Signature of Circulating miR as a Tool to Aid Diagnosis of Primary Brain Tumors in Adults (MIR)	Brain tumors	NA	cfmiR	Blood	NCT03630861
Evaluating the Expression Levels of MiR-10b in Patients with Gliomas	Glioma	NA	cfmiR	Blood, CSF	NCT01849952
Blood Biomarker Signature in Glioma	Glioma	NA	cfmiR	Blood	NCT03698201
Gene Expression, Immunological Status and Metabolome in Glioma Patients	Glioma	NA	cfmiR	Blood	NCT01525459
Pilot Phase 2 Study Whole Brain Radiation Therapy with Simultaneous Integrated Boost for Patients with Brain Metastases	Brain metastasis from solid tumor	Phase II	cfmiR	Blood	NCT03189381
Preventative Trial of DFMO in Patients with High Risk Neuroblastoma in Remission	Neuroblastoma	Phase II	cfmiR	Blood	NCT01586260
Preventative Trial of Difluoromethylornithine (DFMO) in High Risk Patients with Neuroblastoma That is in Remission	Neuroblastoma	Phase II	cfmiR	Blood	NCT02395666
MiRs in Patients with Neurofibromatosis Type 1	NF-1	NA	cfmiR	Blood	NCT01595139
Tertiary Prevention of Head and Neck Cancer with a Dietary Intervention (DietINT)	H&N cancer	Phase II	cfmiR	Blood, saliva	NCT02869399
The Role of miR-29b in the Oral Squamous Cell Carcinoma (miR-29b)	Oral cancer	NA	cfmiR	Blood, saliva	NCT02009852

Innovative Approach to Triage Oral Precancer (iTOP)	Oral cancer	NA	cfmiR	Blood	NCT03202810
Cancer Chemoprevention by Metformin Hydrochloride Compared to Placebo in Oral Potentially Malignant Lesions	Oral cancer	Phase IV	cfmiR	Saliva	NCT03684707
Cancer Chemoprevention by Metformin Hydrochloride in Oral Potentially Malignant Lesions	Oral cancer	Phase III	cfmiR	Saliva	NCT03685409
Molecular Profile of Metastatic Sporadic Medullary Thyroid Cancer Patients and Correlation with Vandetanib	Thyroid cancer	NA	cfmiR	Blood	NCT02268734
Biomarkers to Distinguish Benign from Malignant Thyroid Neoplasm	Thyroid cancer	NA	cfmiR	Blood	NCT01433809
Circulating miRs as Disease Markers in Pediatric Cancers	Pediatric tumors	NA	cfmiR	Blood, CSF	NCT01541800
Longitudinal Study of Biomarkers	Pediatric tumors	NA	cfmiR	Blood, CSF, urine	NCT01595126
Approaches to Identify Early Biomarkers and Pathogenesis of Anthracycline Cardiotoxicity	Pediatric tumors	NA	cfmiR	Blood	NCT04036045
Defining Late Onset Occult Asymptomatic Cardiotoxicity in Childhood Cancer Survivors Exposed to Anthracycline Therapy	Pediatric tumors	NA	cfmiR	Blood	NCT01671696
Blood and Cerebrospinal Fluid for Pediatric Brain Tumor Research	Pediatric CNS tumors	NA	cfmiR	Blood, CSF	NCT01556178
Natural Berry Extract Treatment of Hemangiomas (Pediaberry)	Hemangioma	Phase I	cfmiR	Urine	NCT04020419
Discovery of Sirolimus Sensitive Biomarkers in Blood	Lymphangioleio myomatosis	NA	cfmiR	Blood, urine	NCT03304678
Volumetric Intensity Modulated Arc Therapy vs. Conventional Radiotherapy for Cancer Pain	Painful cancer metastasis	Phase II	cfmiR	Blood	NCT03374592
Effect of Oral Propranolol on mRNA Expression in Symptomatic Cavernous Malformation	Cavernous Malformation	Phase II	cfmiR	Blood	NCT03474614

Abbreviations: NA, Not Available; NSCLC, Non-Small-Cell Lung Cancer; BAL, Bronchoalveolar Lavage; MPM, Malignant Pleural Mesothelioma; CCRT, Chemoradiotherapy; HCC, Hepatocellular Carcinoma; RCC, Renal Cell Carcinoma; AML, Acute Myeloid Leukemia; B-ALL, B-cell Acute Lymphoblastic Leukemia; DFMO, Difluoromethylornithine; H&N, Head and Neck Cancer; CSF, cerebrospinal fluid.
